# Overexpression of *UGPase* and *SPP* genes in *Nicotiana tabacum* leaves causes accelerated plant development and increased biomass

**DOI:** 10.5114/bta/201460

**Published:** 2025-03-31

**Authors:** Magdalena Rakoczy, Jan Podkowinski, Marek Figlerowicz

**Affiliations:** Institute of Bioorganic Chemistry PAS, Poznan, Poland

**Keywords:** energy value, leaf-specific promoter, *Nicotiana tabacum*, sucrose phosphate phosphatase, sucrose synthesis, uridine diphosphate-glucose pyrophosphorylase

## Abstract

**Background:**

Sucrose phosphate phosphatase (*SPP*) and uridine diphosphate-glucose pyrophosphorylase (*UGPase*) genes were overexpressed in *Nicotiana tabacum* to enhance the efficiency of the photosynthesis-related sucrose synthesis pathway, the primary route for incorporating newly fixed carbon into plant metabolism.

**Materials and methods:**

To target transgene expression specifically to the leaves, the *Chrysanthemum x morifolium* Rubisco small subunit promoter was used.

**Results:**

Transgenic plants overexpressing *HvSPP* and *HvUGPase* exhibited high transgene expression in the leaves, exceeding those of the corresponding *N. tabacum* genes by more than tenfold. These plants grew faster and entered the generative phase earlier than control plants, without showing any other developmental abnormalities. By the end of the generative phase, transgenic plants had greater dry mass and contained a higher proportion of carbohydrates than the control group. In result, they accumulated 14.9–17.5% more energy in the aboveground parts compared to reference plants.

**Conclusions:**

The high leaf specificity of the *C. x morifolium* Rubisco small subunit promoter was confirmed, indicating that transgene activity in leaves was effectively separated from its effects on metabolism in non-photosynthetic tissues. Overexpression of *HvUGPase* and *HvSPP* under this promoter accelerated plant growth and development, ultimately increasing biomass. These characteristics are particularly advantageous for energy crops grown as after-crops or in regions with short growing seasons.

## Introduction

Bioenergy and biofuels are rapidly expanding sectors of the global economy. In the European Union, approximately half of renewable energy, including biofuels, is derived from plant material, with similar trends observed in other regions (Wozniak and Twardowski, 2018; Reid et al., 2020; Antar et al., 2021). Plants used as feedstock for bioenergy production are the focus of breeding and research efforts aimed at improving their usability. These studies target both the identification of new species with high productivity and low soil requirements and the genetic modification of known crop species to enhance biomass production efficiency or increase the energy value of plant material (Li and Chen, 2018; Kluts et al., 2019; Malik, 2023).

**Figure 1:**
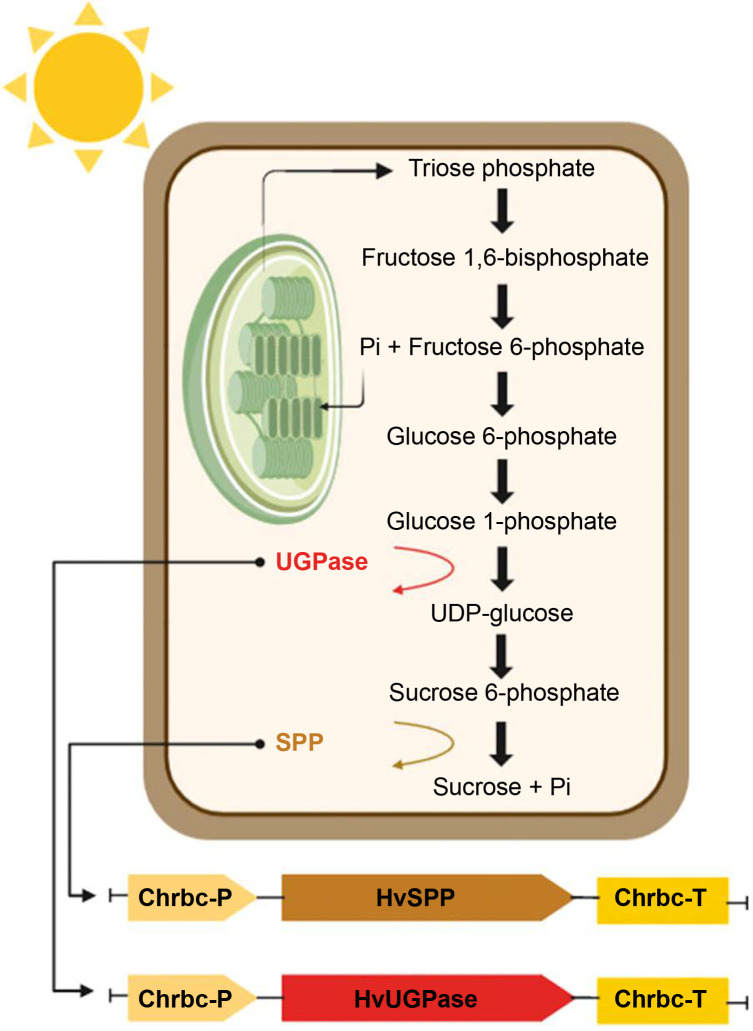
Photosynthesis-related sucrose synthesis pathway, enzymes UGPase (ochre), and SPP (light brown) are shown, Pi – inorganic phosphate

The photosynthesis-related sucrose synthesis pathway, the primary route for incorporating newly fixed carbon into plant metabolism, is a key focus of such research (Margaritopoulou et al., 2016; Nowicka et al., 2018; Liu et al., 2021; Duff and Kretzmer, 2023). This pathway, located in the cytoplasm of mesophyll cells, is linked to carbon dioxide fixation, triose phosphate transport, and sucrose concentration (Huber and Huber, 1992; Lunn and MacRae, 2003; Kleczkowski et al., 2004). Plant productivity and development depend on the activity of enzymes involved in the final stages of this pathway: uridine diphosphate-glucose pyrophosphorylase (UGPase), sucrose phosphate synthase (SPS), and sucrose phosphate phosphatase (SPP) (Fig. 1) (Li et al., 2014; Maloney et al., 2015; Falter and Voigt, 2016; Anur et al., 2020). These enzymes play roles not only in sucrose synthesis but also in its breakdown during transport, sequestration, and sugar metabolism in accumulating organs (Park et al., 2010; Bihmidine et al., 2013; ElSayed et al., 2017; Duan et al., 2021).

Consequently, overexpressing these enzyme genes under a strong ectopic promoter does not differentiate between enhanced efficiency of the photosynthesis-related sucrose synthesis pathway and increased sucrose unloading in consuming or accumulating organs (Coleman et al., 2007; Payyavula et al., 2014). Additionally, ectopic promoters prevent the targeted sequestration of sucrose in selected organs or metabolic processes of energy crops.

Thus, this study aimed to investigate how increased expression of *HvUGPase* and *HvSPP* under a leafspecific promoter influences plant productivity and biomass energy value. A strong promoter derived from the small Rubisco subunit of *Chrysanthemum × morifolium* was used in *Nicotiana tabacum*, a model energy plant in which cell walls serve as the primary carbon sink (Outchkourov et al., 2003; Barla and Kumar, 2019).

## Materials and methods

### pB-Chrbc binary vector and constructs assembly

The cassette containing the *ChrbcS1* promoter and terminator was designed based on the sequence of the ribulose-1,5-bisphosphate carboxylase small subunit gene (AY163904) (Outchkourov et al., 2003). Synthetic DNA fragments *Chrbc-P* and *Chrbc-T*, corresponding to nucleotide positions 1–1013 bp (promoter and 5′ UTR) and 1724–2658 bp (terminator with 3′ UTR), respectively, were synthesized with minor modifications to incorporate restriction sites (*Pst*I, *Avr*II, *Spe*I, *Stu*I, and *EcoR*I). These fragments were assembled into the pEX-A2 vector. The cassette was then transferred into the pBI121 vector (Chen et al., 2003) using *HindIII* and *EcoRI* digestion followed by ligation, resulting in the pBChrbc vector. Integration of the *HindIII–EcoRI ChrbcS1* cassette into the vector was confirmed by restriction enzyme digestion and sequencing.

**Figure 2:**
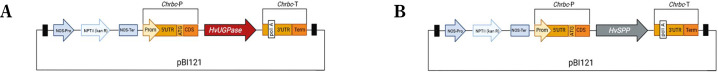
Scheme of genetic constructs: (**A**) pB-Chrbc-*HvUGPase* construct for HvUGPase overexpression, (**B**) pB-Chrbc-*HvSPP* construct for HvSPP overexpression. The synthetic fragments Chrbc-P and Chrbc-T are shown: the Chrbc-P fragment containing the ChrbcS1 promoter region (Prom) with 5′ UTR and the first three codons (ATG, CDS), and the Chrbc-T fragment containing the ChrbcS1 terminator with 3′ UTR and poly A signal (poli A); black boxes – T-DNA left and right border regions; Nos-Pro (navy blue) – nopaline synthase promoter, NPTII (pale blue) – neomycin phosphotransferase II, NOS-Ter (navy blue) **–** nopaline synthase terminator

Transgenes encoding HvUGPase (MN584934) and HvSPP (MN584936) were obtained via reverse transcription PCR (RT-PCR) using RNA extracted from the leaves of *Hordeum vulgare* var. *apex* as a template. The primers used were MZ38 and MZ39 for *HvUGPase* and MZ42 and MZ43 for *HvSPP* (Table 1). The PCR products were digested with *AvrII* and *StuI* or *SpeI*, then ligated into the pB-Chrbc vector, generating the pB-Chrbc-*HvUGPase* and pB-Chrbc-*HvSPP* constructs (Fig. 2). The constructs were verified by restriction enzyme digestion and sequencing.

**Table 1:** Primers used in this study. Primer sequence – primer sequence in 5′ to 3′ orientation, lowercase letters correspond to linkers containing restriction enzyme sites. Range – range of primer nucleotide coordinates corresponding to the sequence used to design the primer. Accession number – GenBank accession and version number of the sequence used to design the primer. Tm – calculated basic Tm of the primer. PCR cycles – number of PCR cycles used to amplify the amplicon in PCR reaction, values without brackets refer to standard PCR reaction, values in brackets refer to ddPCR

Primer ID	Primer sequence	Range [nt]	Accession number	Tm [°C]	PCR cycles
Mz38	5’-aaacctgagg--TGGCCGCCGCCGCCG-3’	11–25	MN584934.1	69	33
Mz39	5’-tttactagt--GGTTTCTGGGGATGCGAG-3’	10–27	MN584934.1	60	33
Mz42	5’-gtgcctagg--ATGGATAAGGTCAAGGGCTCTGC-3’	10–32	MN584936.1	67	35
Mz43	5’-tttaggcct--CTAGTATATGTATGTGTCTGGAGCAC-3’	10–36	MN584936.1	63	35
s-117	5’-TGAACATGGCATCGTGGTGA-3’	1–20	AF485783.1	52	35
s-118	5’-GCTAACGTATCCACGCCGTA-3’	1–20	AF485783.1	54	35
Mz100	5’-GTCCAGCTCTTGGAGATTGC-3’	1–20	MN584934.1	54	33 (40)
Mz101	5’-TGCGGTTTCTAGCTGTAGCA-3’	1–20	MN584934.1	52	33 (40)
Mz104	5’-ATGTTTTCCCTGCTTTGGTG-3’	1–20	EB451958.1	50	(40)
Mz105	5’-TTGGTGTAACCTCCATGCAA-3’	1–20	EB451958.1	50	(40)
Mz108	5’-CTCCAGCCAGACACAGAACA-3’	1–20	MN584936.1	54	33 (40)
Mz109	5’-GTGTTTTTCCGCATGAACCT-3’	1–20	MN584936.1	50	33 (40)
Mz114	5’-TGGTGTCGAGAAATCCCTTC-3’	1–20	FG194480.1	52	(40)
Mz115	5’-CCATCCGCGACAGTCTTATT-3’	1–20	FG194480.1	52	(40)
Mz116	5’-AGCTAAAGCTGATCCGTCCA-3’	1–20	L18908.1	52	33 (40)
Mz117	5’-TTGCAGACTCTGTGGTGAGG-3’	1–20	L18908.1	54	33 (40)

The reference construct, pB-Ref, was derived from the pBI121 vector, in which the CaMV 35S promoter was replaced with a non-functional DNA fragment lacking promoter activity. This fragment corresponded to nucleotide positions 4,070,818–4,071,597 bp of chromosome 10 in *Sorghum bicolor* (Phytozome, *S. bicolor* genome v3.1.1). The pB-Ref construct was verified by restriction enzyme digestion and sequencing.

### Nicotiana tabacum transformation, breeding and screening

*Nicotiana tabacum* cv. W38 plants were transformed with *Agrobacterium tumefaciens* LBA4404 carrying either the pB-Chrbc-*HvUGPase* or pB-Chrbc-*HvSPP* construct using the leaf disc inoculation method (Horsch et al., 1985). Explants were maintained by transferring apical shoot-forming regions to Murashige and Skoog (MS) medium supplemented with carbenicillin (500 mg/l), kanamycin (50 mg/l), and 2% sucrose in plant culture vessels. They were grown in a chamber under a 16 h light/8 h dark photoperiod at 25°C until resistant adventitious buds formed. These buds were transferred to the same medium to select rooted, resistant plants.

Regenerated plantlets with roots, grown *in vitro* for 5–6 weeks, were screened for transgene expression via RT-PCR using specific primers: MZ100 and MZ101 for *HvUGPase* and MZ108 and MZ109 for *HvSPP* (Table 1). T_0_ plants expressing the transgene were transferred to a perlite : sand (1:1) mixture and grown in a greenhouse without a selection agent. Plants were watered twice a week, first with tap water and then with an MS solution to ensure proper nutrition. Transgene expression was retested at the onset of flowering using RNA extracted from leaves cultivated without a selection agent. The plants were then grown to seed maturation.

T_1_ seeds were germinated on MS medium supplemented as described above and then transferred to perlite : sand (1:1) mixture, where they were grown without a selection agent. Cultivation was carried out in a growth chamber under a 16 h light/8 h dark photoperiod at 24°C and 65% relative humidity. Plants were watered twice a week, first with tap water and then with an MS solution to ensure proper nutrition. During growth without selection, T_1_ plants were tested for transgene expression first at the 4–6 leaf stage and again at the onset of flowering. RNA for these analyses was isolated from leaves cultivated without a selection agent. Phenotypic characteristics of T_1_ transgenic plants were recorded twice a week, including plant height (measured from the root collar to the apex), the length of the three longest leaves, and the presence of flower buds or flowers. At 69 days after potting (DAP), the entire aerial part of the plant was harvested and analyzed for total dry mass, lignin, cellulose, and soluble carbohydrate content (see: *Materials and methods, Cell wall analysis — determination of mono- and disaccharides, cellulose, hemicellulose, and lignin*), as well as energy value (see: *Materials and methods, Determination of the energy value of N. tabacum plants*).

Plants transformed with the pB-Ref construct were used as reference plants to exclude the possibility that observed effects in the plants of interest resulted from transformation and regeneration processes or the presence of the vector itself (Payyavula et al., 2014; Victorathisayam et al., 2022; Zhang et al., 2022). Transformation and breeding were performed as described above, and reference plants were screened for the presence of the *GUS* gene in genomic DNA via PCR using primers s-117 and s-118 (Table 1).

RT-PCR analyses confirmed that reference plants did not exhibit transgene expression at any stage of breeding. Two independent reference plant lineages were identified: lineage no. 34 and lineage no. 35. T_1_ plants from these lineages were grown in parallel with the experimental plants as controls, with two T_1_ plants from lineage no. 34 and three from lineage no. 35.

### Transgenic plant validation with PCR and RT-PCR

Total RNA was isolated from plant material using the RNeasy Plant Kit (Qiagen) and digested with RNase-free DNase I (Thermo Scientific). Reverse transcription (RT) was performed using the Maxima First Strand cDNA Synthesis Kit for RT-qPCR (Thermo Scientific) with 0.2 μg/μl RNA. Aliquots of the RT reaction were used directly as templates for PCR amplification in a 15.0 μl final volume containing 0.025 U/μl *Taq* DNA polymerase (Thermo Scientific), 1.75 mM MgCl_2_, 0.2 mM dNTPs, and 0.4 μM forward and reverse primers (see above).

PCR conditions were as follows: initial denaturation at 95°C for 2 min, followed by cycles of 94°C for 30 s, 56°C for 30 s, and 72°C for 50 s, with a final extension at 72°C for 5 min. The number of PCR cycles varied depending on the amplified gene. Genomic DNA was digested with RNase A (Thermo Scientific), extracted with phenol and chloroform, precipitated with ethanol, and dissolved in TE buffer. PCR amplification of target genes was performed using the same primers and conditions as described above. The *N. tabacum* gene for the L25 ribosomal protein was used as the reference gene for both PCR and RT-PCR analyses (Schmidt and Delaney, 2010). Primers MZ116 and MZ117 for this reference gene were designed based on the L18908 sequence (Table 1).

### Droplet digital PCR

The ddPCR (droplet digital PCR) reaction contained 1.0 μl of the template (RT reaction diluted with water), 200 nM primers (see above), and 1× QX200 ddPCR EvaGreen Supermix (Bio-Rad) in a final volume of 20.0 μl. Reactions were partitioned into droplets using the QX200 Droplet Generator (Bio-Rad), and DNA was amplified under the following conditions: initial denaturation at 95°C for 5 min, followed by 40 cycles of 95°C for 30 s, 59°C for 30 s, and 72°C for 45 s, with a final extension at 72°C for 2 min. Signal stabilization was performed at 4°C for 5 min, followed by 90°C for 5 min.

The fluorescence amplitude of the generated droplets was measured using a QX100 droplet reader (BioRad), and data were analyzed with QuantaSoft (BioRad) software. The *N. tabacum* gene for the ribosomal L25 protein was used for expression normalization. Primers MZ104 and MZ105 were used for PCR analysis of *N. tabacum NtUGPase*, while primers MZ114 and MZ115 were used for *NtSPP* (Table 1).

RT-ddPCR was used for quantitative analyses described in section *Results, Chrbc-P promoter activity*, i.e.: 1 – to examine the activity of the *Chrbc-P* promoter in leaves before the completion of the full expansion phase and compare this expression with that in roots of young plants, 2 – to compare transgene expression between young and mature plants, and 3 – to examine the expression of *N. tabacum* genes corresponding to the transgenes.

### Cell wall analysis – determination of mono- and disaccharides, cellulose, hemicellulose, and lignin

The analysis of cell wall composition was performed using the Ankom A200 and Foss Fibertec 2010 Auto Fiber Analysis System (FOSS Analytical AB, Sweden). The principle of the analysis is based on the separation of the analyzed material into fractions, named according to chemical analytical techniques: crude fiber (CF), neutral detergent fiber (NDF), acid detergent fiber (ADF), and acid detergent lignin (ADL). These techniques involve sequential chemical treatments to solubilize the nonfiber components of the analyzed material, followed by the determination of the remaining residue. Briefly, the CF method consists of sequential treatment of the analyzed material with hot sulfuric acid and hot sodium hydroxide, producing residues composed of cellulose (50–80% d.m.), hemicellulose (approx. 20% d.m.) and lignin (10–50% d.m.). The NDF method treats the material with a neutral detergent solution, leaving a residue composed of cellulose, hemicellulose, and lignin. The ADF method involves treatment with an acid detergent solution, yielding residues consisting of cellulose and lignin. The ADL method further extracts the ADF residue with sulfuric acid, isolating lignin. Detailed protocols for these methods are provided in the Application Notes (FOSS Analytical AB) included in the Supplementary Files: AN_304, AN3429, AN3432, and AN3434.

*N. tabacum* stem segments and leaves were dried at 72°C, ground using an IKA MF10 grinder into particles smaller than 1.0 mm, and sieved to obtain a fraction with a grain size of 1.0–0.5 mm. Humidity was determined using a Binder FD53 drying oven according to ISO 18134. The hot water-soluble substances, NDF, ADF, and ADL were measured using the Ankom A200 and Foss Fibertec 2010 Auto Fiber Analysis System (FOSS Analytical AB, Sweden) according to the manufacturer’s protocols: Foss AN3434 (compliant with ISO 16472) and Foss AN3429 (compliant with ISO 13906). The content of lignin, cellulose, and hemicellulose in the plant material was calculated as follows: hemicellulose = NDF – ADF; cellulose = ADF – ADL; and lignin = ADL. Calculations were performed following the Foss AN_304 protocol. All determinations were conducted in triplicate.

For the analysis of mono- and disaccharides, the plant material was further ground using a Foss Cyclotec 1093 mill to obtain particles smaller than 0.25 mm. The determination of soluble carbohydrate content was carried out using a spectrophotometric method with acid hydrolysis performed using 3,5-dinitrosalicylic acid (Sigma D0550). The ground plant material was extracted in ddH_2_O at *T* = 45°C for 1 h under a reflux condenser. The solution was left to stand for 24 h, then filtered and adjusted to a uniform volume. The pH was stabilized to 7.0–7.5. The reactions with 3,5-dinitrosalicylic acid were performed at *T* = 75°C for 5 min, after which the sample was cooled in water at *T* = 5°C. A Helios Gamma spectrophotometer was used for the analyses, with measurements taken at a wavelength of 540 nm. Anhydrous glucose (Sigma G5767) was used to prepare the standard curve. All determinations were conducted in triplicate. Detailed FOSS protocols referenced above are provided in the Supplementary Materials.

### Determination of the energy value of Nicotiana tabacum plants

*N. tabacum* stem segments and leaves were dried at 72°C and ground as above into grains below 1.0 mm. Binder FD53 drying oven was used to determine the humidity according to the ISO 18134 standard. The gross calorific value (GCV) of the plant material was measured at a constant volume and a reference temperature of 25°C using an IKA C2000 combustion calorimeter. The bomb calorimeter was calibrated by the combustion of certified benzoic acid in accordance with ISO 18125 (PN-EN ISO 18125). The determination of energy value was conducted at the Department of Plant Breeding and Seed Science, University of Warmia and Mazury. All assays were performed in triplicate.

Data on the energy value and dry mass of the aboveground part of each plant were used to calculate the total energy sequestered by the above-ground part of the given plant. This value was obtained as the product of the energy value and the dry mass of the aboveground part of the given plant. The formula for calculating the total energy sequestered by the aboveground biomass is provided below:

Total energy sequestered by the aboveground part of the plant = Energy value × Dry mass of the aboveground part of the given plant.

### Statistical analysis and data presentation

Statistical analysis of the presented data was conducted using GraphPad Prism 10 software. An unpaired *t*-test was used to assess the significance of differences between groups. Results are presented as mean ± standard deviation (SD), with statistical relevance determined at *p* < 0.05. Asterisks indicate the level of significance: *p < 0.05, **p < 0.01, and ***p < 0.001.

All graphs in this publication were generated using GraphPad Prism. Data are presented as mean ± SD, with error bars representing the SD. Figures 1 and 2 were created using BioRender.

## Results and discussion

### Transgenic plant preparation

**Figure 3:**
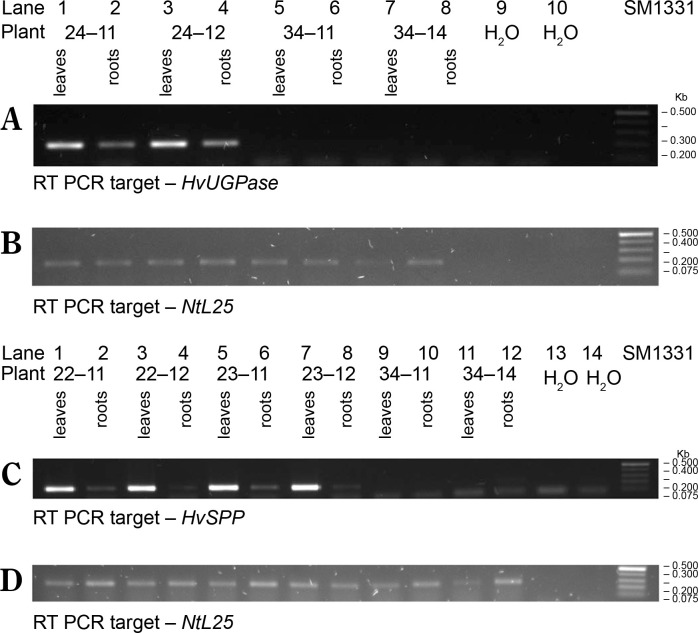
RT-PCR analysis of the T1 transgenic plants at 4 weeks after sowing, a different number of cycles were used for individual genes, therefore the intensity of the bands can be compared only within a given gel. The plants with *HvUGPase* expression: (**A**) PCR targeted to *HvUGPase* transgene, (**B**) PCR targeted to *NtL25*; **A** and **B** lanes: 1) plant 24–11 – leaves, 2) plant 24–11 – roots, 3) plant 24–12 – leaves, 4) plant 24–12 – roots, 5) reference plant leaves 34–11, 6) reference plant roots 34–11, 7) reference plant leaves 34–14, 8) reference plant roots 34–14, 9 and 10 – no template control. The plants with *HvSPP* expression: (**C**) PCR targeted to *HvSPP* transgene, (**D**) PCR targeted to *NtL25*; **C** and **D** lanes: 1) plant 22–11 – leaves, 2) plant 22–11 – roots, 3) plant 22–12 – leaves, 4) plant 22–12 – roots, 5) plant 23–11 – leaves, 6) plant 23–11 – roots, 7) plant 23–12 – leaves, 8) plant 23–12 – roots, 9) reference plant leaves – 34–11, 10) reference plants roots – 34–11, 11) reference plant leaves – 34–14, 12) reference plants roots – 34–14, 13 and 14) no template control

Transgenic *N. tabacum* plants carrying the pB-Chrbc-*HvSPP* and pB-Chrbc-*HvUGPase* constructs were generated via *Agrobacterium*-mediated transformation. Transformants were examined using RT-PCR, which identified lineages with high transgene overexpression: lineage no. 24 for plants with the *HvUGPase* transgene and lineages no. 22 and no. 23 for plants with the *HvSPP* transgene. Two T_1_ plants from each of these lineages were selected for further analysis. During breeding without a selection factor, transgene presence and activity in these plants were assessed twice, first at 4 weeks postsowing and again at the onset of flowering (Fig. 3). No transgene loss was observed during T_1_ plant cultivation.

**Figure 4:**
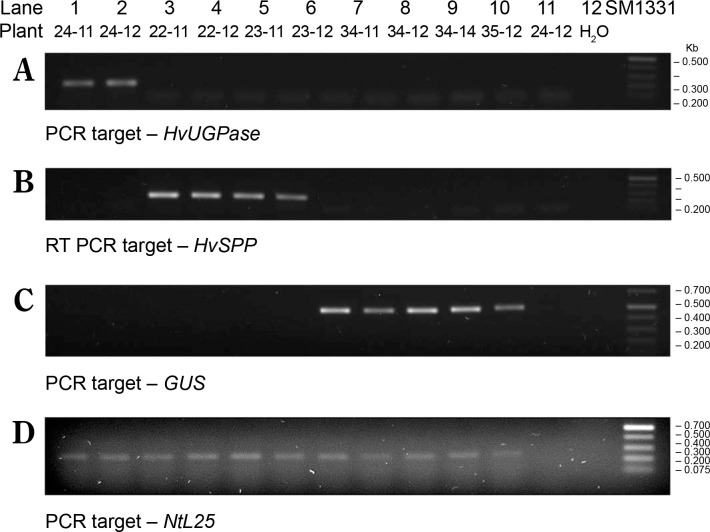
PCR identification of the transgene in the genomic DNA of leaves of T1 transgenic plants 4 weeks after sowing, a different number of cycles were used for individual genes, therefore the intensity of the bands can be compared only within a given gel. (**A**) PCR targeted to *HvUGPase*, (**B**) PCR targeted to *HvSPP*, (**C**) PCR targeted to *GUS*, and (**D**) PCR targeted to *NtL25*. Lanes in **A–D**: 1) *HvUGPase* plant 24–11, 2) *HvUGPase* plant 24–12, 3) *HvSPP* plant 22–11, 4) *HvSPP* plant 22–12, 5) *HvSPP* plant 23–11, 6) *HvSPP* plant 23–12, 7) reference plant 34–11, 8) reference plant 34–12, 9) reference plant 34–14, 10) reference plant 35–11, 11) reference plant 35–12, and 12) no template control

Reference plants transformed with the pB-Ref vector carrying the nonexpressed *GUS* gene were prepared following the same procedure. During selection and breeding, reference plants were analyzed by PCR amplification using genomic DNA as a template (Fig. 4). Although the *GUS* gene was detected in genomic DNA, RT-PCR analyses revealed the absence of transgene expression. Plants transformed with the nonexpressed gene were used as references to exclude the possibility that observed effects in the experimental plants resulted from transformation, regeneration, or the presence of the transgene cassette itself (Payyavula et al., 2014; Victorathisayam et al., 2022; Zhang et al., 2022). Two reference plant lineages, no. 34 and no. 35, were identified. Their T_1_ progeny was cultivated alongside *HvUGPase*- and *HvSPP*- expressing plants and used as references.

### Chrbc-P promoter activity

**Figure 5:**
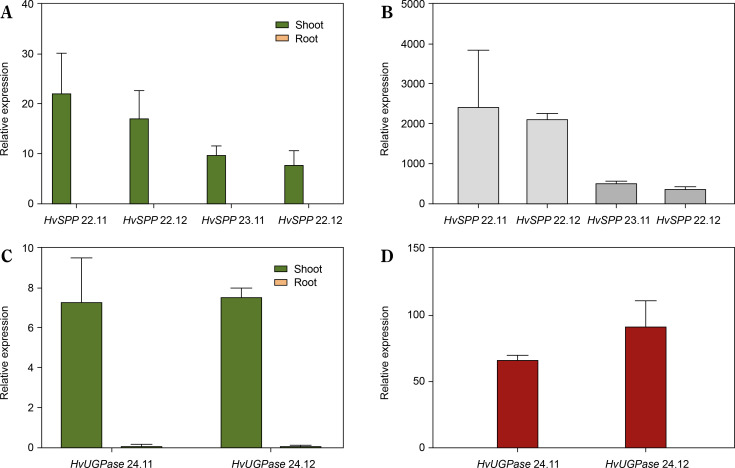
Relative transgene expression in T1 transgenic plants, 4 weeks after sowing: green – relative expression in leaves before the end of the expansion phase, orange – relative expression in roots, values below 0.1 are not visible. (**A**) Relative expression of *HvSPP* in young leaves and roots of transgenic plants, the relative expression of *HvSPP* in roots is below 0.1. (**B**) Leaf-to-root ratio of *HvSPP* relative expression. (**C**) Relative expression of *HvUGPase* in young leaves and roots. (**D**) Leaf-to-root ratio of *HvUGPase* relative expression. Data are presented as mean ± standard deviation (SD) from two technical repetitions

The relative expression of transgenes in leaves and roots was analyzed using reverse transcription followed by digital droplet PCR (RT-ddPCR), with the L25 ribosomal protein gene as the reference (Fig. 5). In *HvSPP* transgenic plants, relative transgene expression in young, developing leaves at 4 weeks after sowing ranged from 7.8 to 22.1 and was 387 to 2275 times higher than in roots. In *HvUGPase* overexpressing plants, relative expression in young leaves ranged from 7.3 to 7.6 and was 67 to 90 times higher than in roots. No transgene expression was detected in any of the five reference plants. RT-ddPCR analysis indicated that in young leaves, the *Chrbc-P* promoter exhibited higher activity than the L25 ribosomal protein gene promoter.

**Figure 6:**
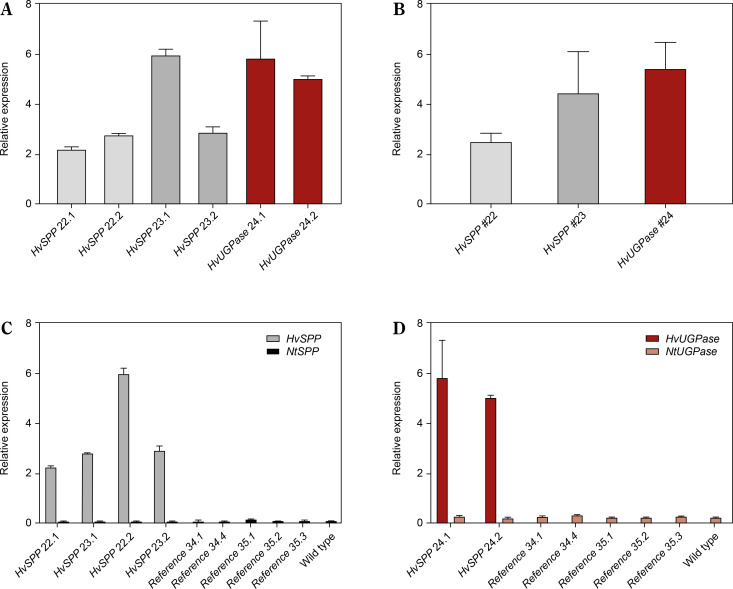
Relative expression of transgenes and *Nicotiana tabacum* genes in T1 leaves after the end of the expansion phase: grey – a relative expression of *HvSPP*, ochre – a relative expression of *HvUGPase*, black – a relative expression of *NtSPP*, pink – a relative expression of *NtUGPase*. (**A**) Relative expression of *HvSPP* and *HvUGPase*. (**B**) Average relative expression of the transgenes in transgenic lines. (**C**) Relative expression of *HvSPP* and *N. tabacum NtSPP* in transgenic, reference, and wild-type plants. (**D**) Relative expression of *HvUGPase* and *N. tabacum NtUGPase* in transgenic, reference, and wild-type plants. Data are presented as mean ± standard deviation (SD) from two technical repetitions

The activity of the *Chrbc-P* promoter decreased during plant development. In *HvSPP* transgenic plants, relative transgene expression in mature leaves after seed set was 2.0 to 7.8 times lower than in young, developing leaves at 4 weeks after sowing (Fig. 6). Similarly, in *HvUGPase* overexpressing plants, transgene expression in mature leaves decreased 1.4-fold during development. Despite the reduced activity of the *Chrbc-P* promoter, transgene expression in mature leaves remained significantly higher than that of the corresponding *N. tabacum* genes — 33.1- to 56.4-fold for *HvSPP* and 14.8- to 20.9-fold for *HvUGPase* (Table 2). Notably, this high transgene activity did not affect the expression of endogenous *N. tabacum SPP* and *UGPase* genes, which remained comparable between transgenic and reference plants.

**Table 2:** Relative expression of the transgenes – *HvUGPase* and *HvSPP*, and *Nicotiana tabacum* genes – *NtUGPase* and *NtSPP* – in mature leaves of T1 transgenic *N. tabacum* plants

Plants	Relative expression of transgene in mature leaves	Relative expression of *N. tabacum* gene in mature leaves
T1 HvSPP plants, lineage #22	Transgene – *HvSPP*	*N. tabacum* gene – *NtSPP*
	2.24–2.80	0.068–0.068
T1 HvSPP plants, lineage #23	Transgene – *HvSPP*	*N. tabacum* gene – *NtSPP*
	2.91–5.98	0.088–0.106
T1 reference plants, lineage #34 and #35	Transgene – *HvSPP*	*N. tabacum* gene – *NtSPP*
	Not detected	0.083–0.115
T1 HvUGPase plants, lineage #24	Transgene – *HvUGPase*	*N. tabacum* gene – *NtUGPase*
	5.04–5.86	0.280–0.342
T1 reference plants, lineage #34 and #35	Transgene – *HvUGPase*	*N. tabacum* gene – *NtUGPase*
	Not detected	0.243–0.320

**Figure 7:**
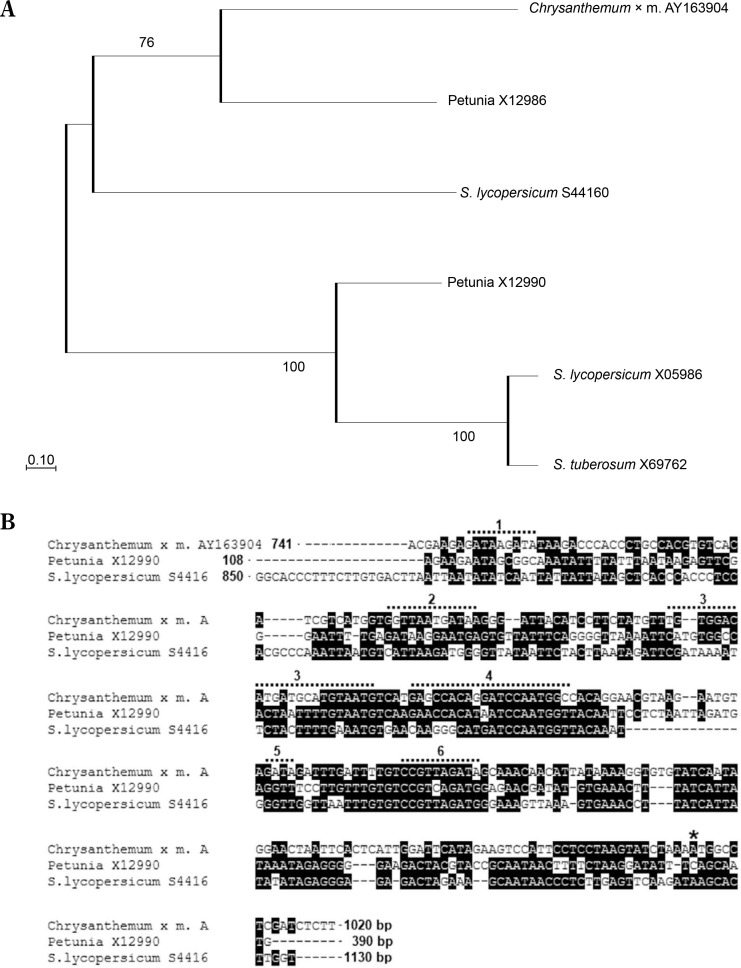
Conservancy and functional motifs in the proximal region of the *rbcS1* promoter from *Chrysanthemum × morifolium* (AY163904). The proximal region of the *rbcs* promoters from *C.* × *morifolium* (AY163904) and *Solanaceae* plants was aligned with ClustalW, and a Maximum Likelihood phylogenetic tree with 1000 bootstrap replicates was generated using MEGA11. (**A**) Phylogenetic analysis shows close clustering between the *rbcS* promoter from *C.* × *morifolium –* AY163904, and two of the *Solanaceae* promoters: X12986 from petunia and S44160 from *S. lycopersicum*. These two *rbcS* promoters from petunia and *S. lycopersicum* are more similar to the *rbcS1* promoter from the *C.* × *morifolium* than to other *rbcS* promoters from *Solanaceae*. (**B**) Alignment of *rbc*S promoters from: *C.* × *morifolium* (X12986), petunia (X12986), and tomato (S44160), positions with more than 60% conservation are highlighted in black, coordinates of analyzed sequences are given according to GenBank. Regions with cis-acting regulatory elements in the proximal region of *C.* × *morifolium rbcS1* promoter AY163904 are marked with a dotted line (top line): 1 – light-regulated G-box (PLACE, S000041) and IBOX CORE (PLACE, S000199); 2 – light-regulated G-box (PLACE, S000041), IBOX CORE (PLACE, S000199) and SURE; 3 – GT1 Consensus (PLACE, S000198); 4 – CAAT BOX (PLACE, S000030) and GATA-box (PLACE, S000039); 5 – light-regulated G-box (PLACE, S000041); 6 – GATA-box (PLACE, S000039) and CAAT BOX (PLACE, S000030). The promoter regions used in A and B: AY163904.1 – *C.* × *morifolium*, 741–1020 bp; X05986 – *S. lycopersicum*, 54-340 bp; X69762 – *S. tuberosum*, 180-464 bp; X12990 – *Petunia x hybrida*, 85-351 bp; X12986 – *Petunia x hybrida*, 108-390; S44160 – *S. lycopersicum*, 850-1130 bp. The start codon in the *Chrysanthemum* AY163904 sequence is marked with an asterisk

The observed decrease in *rbcS* promoter activity following leaf expansion may be linked to reduced carbon compound demand at this developmental stage, a pattern previously reported for *rbcS* genes in cotton, banana, rice, and other plants (Thomas-Hall et al., 2007; Suzuki et al., 2009; Parishot et al., 2013). Similarly, in tomato, all five *rbcS* genes highly expressed in leaves are developmentally regulated (Sugita and Gruissem, 1987). The high efficiency of the *Chrbc-P* promoter in young *N. tabacum* leaves was previously demonstrated by Outchkourov et al. (2003). Here, RT-ddPCR analysis showed that also in mature leaves of *N. tabacum* this promoter has an activity higher than the ribosomal protein L25 promoter. Furthermore, this study experimentally validated the highly leaf-specific activity of the *Chrbc-P* promoter in driving transgene overexpression. The strong transcriptional activity of this chrysanthemum-derived promoter in *N. tabacum* is likely due to the conservation of numerous regulatory motifs and enhancers, as well as the similarity in their arrangement in *Asteraceae* and *Solanaceae* species (Fig. 7).

### Phenotype of plants with overexpression of HvSPP and HvUGPase

**Figure 8:**
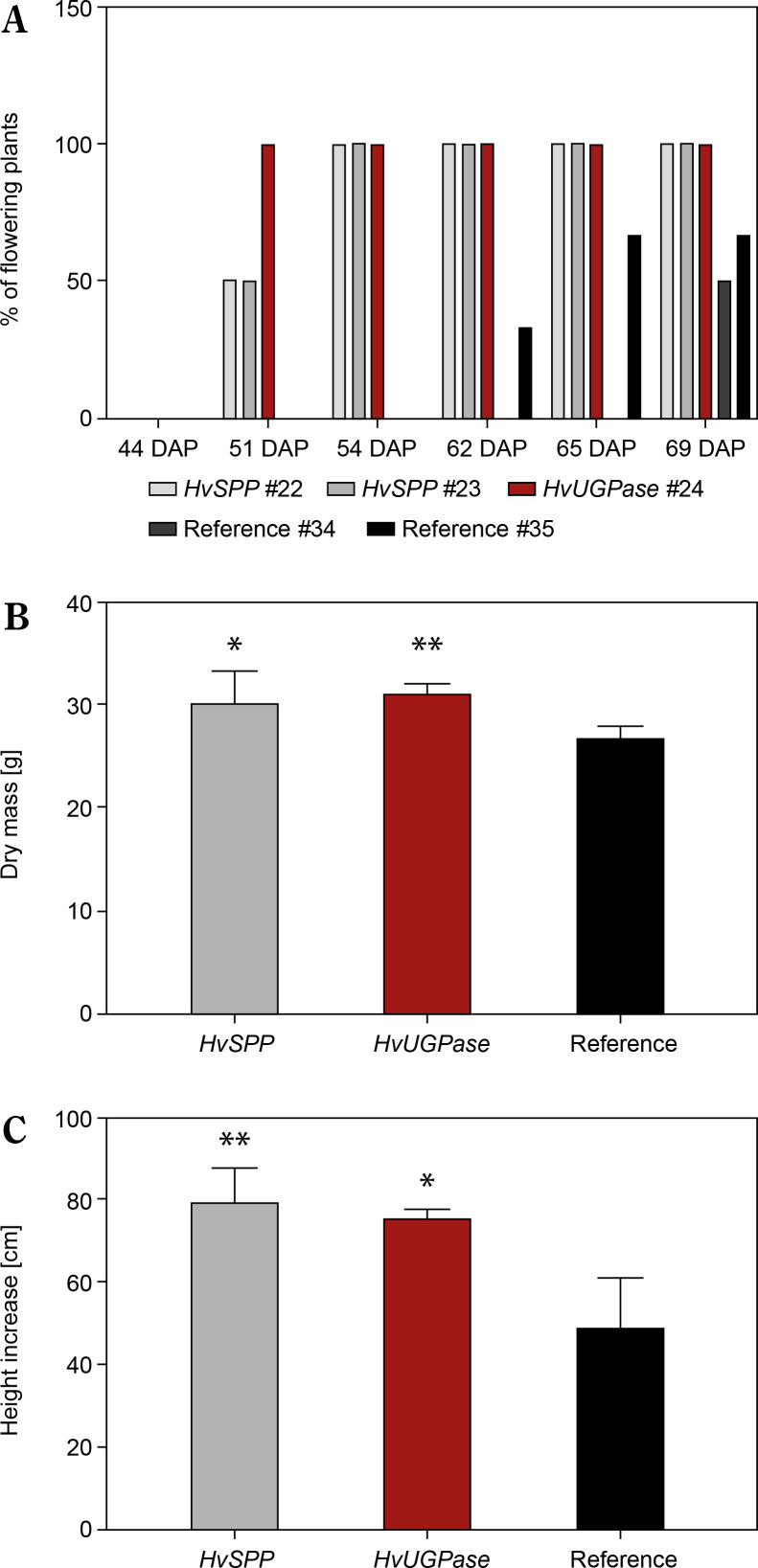
Flowering time, dry mass, and height of T1 plants overexpressing *HvUGPase* and *HvSPP* compared to reference plants. (**A**) Percentage of plants flowering between 30 and 69 DAP. (**B**) Dry mass at 69 DAP. (**C**) Plant height increase from 30 to 69 DAP. (**B** and **D**) Data are presented as mean ± standard deviation (SD) from plants with the given transgene, the asterisks indicate the level of statistical significance, with *p < 0.05, and **p < 0.01

Plants overexpressing *HvSPP* and *HvUGPase* exhibited faster growth and development than reference plants (Fig. 8A, C). Between 30 and 69 DAP, height increased by 41.5% in *HvSPP* overexpressing plants compared to reference plants, while in *HvUGPase* overexpressing plants, the difference was 35.7%. The dry mass (d.m.) of *HvSPP*-overexpressing plants at 69 DAP, when mature seeds were present, was 6% greater than that of reference plants, while in *HvUGPase*-overexpressing plants, this difference was 9% (Fig. 8B). Transgenic plants entered the generative phase earlier, with the first flower buds appearing 11 days earlier than in reference plants. However, no differences were observed in maximum leaf length, leaf width, or flower morphology between transgenic and reference plants.

The association between *UGPase* overexpression and faster growth has been reported in previous studies, though the processes behind this remained unclear due to the use of ectopic promoters. Transgenic plants constitutively overexpressing *UGPase* genes from a variety of organisms – including cotton, jute, sorghum, larch, and even bacteria – exhibited similar phenotypic traits, such as increased vegetative growth, higher dry weight, increased height, and no significant differences in leaf size or area. In *Arabidopsis thaliana*, overexpression of *UGPase* from cotton resulted in increased plant height and growth rate (Wang et al., 2011). Transgenic plants also had higher soluble sugars, starch, and cellulose content, while lignin remained unchanged. A similar phenotype was observed in *A. thaliana* overexpressing *LgUGPase* from larch (*Larix gmelinii*) (Li et al. 2014). The phenotype included enhanced vegetative growth, higher height, increased contents of sucrose and total soluble sugars, as well as higher content of cellulose, and thickened parenchyma cell walls. The content of lignin and hemicellulose was not significantly different between the transgenic lines and WT plants.

In the case of *Sorghum bicolor UGPase* overexpression in *A. thaliana*, only one of five tested transgenes resulted in plants exhibiting the phenotype (Jiang et al., 2015). This outcome was likely due to transcript maturation abnormalities caused by the use of full-length *S. bicolor* genes in *A. thaliana*. Transgenic *A. thaliana* carrying the *Sobic.006G213100* gene encoding *SbUGPase* showed faster root growth than WT plants. Faster growth was observed in young plants, with 15-day-old plants having higher fresh weight, though this difference was no longer present in mature 65-day-old plants. *A. thaliana* overexpressing *SbUGPase* also exhibited accelerated development, with earlier flowering initiation than WT plants.

Similar growth enhancement was observed in *Corchorus capsularis* (jute) overexpressing *CcUGPase*, a much larger plant than *A. thaliana* (Zhang et al., 2013). Transgenic jute plants carrying a *UGPase* transgene from jute showed increased cellulose content while maintaining lignin content comparable to WT plants. In *N. tabacum* transformed with a bacterial *UGPase* gene from *Acetobacter xylinum*, transgenic plants exhibited increased height and internode length, though this did not result in greater stem dry mass (Coleman et al., 2006). Transgenic tobacco with a the transgene encoding bacterial UGPase also showed increased content of total sugars with unchanged content of sucrose. Furthermore, the transgenic plants did not show changes in content of starch and cellulose.

The above examples show that in most cases, regardless of plant species, ectopic overexpression of *UGPase* leads to accelerated growth and development, often accompanied by a shortened vegetative phase and earlier flowering. However, changes in carbohydrate and lignin content vary depending on the plant species, likely due to the dual role of *UGPase*. Beyond its function in the photosynthesis-related sucrose synthesis pathway, UGPase also catalyzes the conversion of uridine triphosphate to uridine diphosphate glucose, which serves as a glucosyl donor in cellulose biosynthesis. Enhanced *UGPase* expression in nonphotosynthetic tissues may increase cellulose synthesis, though the final outcome depends on the physiological processes specific to each plant species.

A relationship between the *SPP* gene and plant height has been demonstrated in *N. tabacum*, where reduced *SPP* expression resulted in decreased growth rates (Chen et al., 2005). However, *A. thaliana* plants overexpressing sorghum *SPP* genes under a constitutive promoter did not show statistical differences from wild-type plants in biomass, height, or flowering time under normal growth conditions (Jiang et al., 2015). In the present study, increased expression of *HvSPP* under the *Chrbc-P* promoter was shown to have a positive effect on plant growth rate and biomass accumulation, producing a phenotype similar to that of *HvUGPase* overexpressing plants under the same promoter. This suggests that both genes likely act through a similar mechanism, most probably linked to their role in the photosynthesis-related sucrose synthesis pathway.

The observed increase in growth rate and biomass accumulation may result from an enhanced supply of sucrose, the primary form in which newly fixed carbon is transported over long distances. In *N. tabacum*, which lacks specific carbohydrate-sequestering organs, excess sucrose is likely utilized for cell wall biosynthesis, contributing to accelerated growth. However, this does not explain why transgenic plants enter the generative phase, bloom, and set seeds earlier than reference plants.

A link between earlier flowering time and the overexpression of genes for enzymes involved in sucrose metabolism has been previously reported (Coleman et al., 2010; Gebril et al., 2015; Jiang et al., 2015; Seger et al., 2015; Wei et al., 2015). Given that accelerated generative phase initiation has been observed in plants overexpressing genes encoding various sugar metabolism enzymes – including sucrose synthase, *UGPase*, and *SPS* – this phenotype may be associated with the role of sugars as signaling molecules. Sugars have been implicated in regulating apical dominance, flowering, seed maturation, and the circadian cycle (Lastdrager et al., 2014; Mason et al., 2014; Philippou et al., 2019; Saksena et al., 2020; Yoon et al., 2021; Guo et al., 2023; Wang et al., 2024).

Faster development and earlier flowering were not observed in transgenic maize plants overexpressing the *SPS* gene, which encodes one of the enzymes in the photosynthesis-related sucrose synthesis pathway, alongside UGPase and SPP (Duff and Kretzmer, 2023). In these maize plants, two mesophyll-specific and light-regulated promoters, similar to the *Chrbc-P* promoter used in the present study, were employed. Transgenic maize expressing *SPS* under both promoters exhibited higher sucrose content and lower starch levels in freshly expanded leaves at the V6 stage. However, these plants did not show an increase in yield, defined as seed mass. Notably, the vegetative mass of whole plants was not measured in that study, making direct comparisons to the present findings difficult.

Another obstacle to such a comparison is the difference between maize, a C4 monocotyledonous plant with Kranz anatomy, and *N. tabacum*, a C3 dicotyledonous plant used in our study because the transgenic plant phenotype is the result of interaction between the transgene product activity and the specific metabolism and physiology of the plant.

### Quantitative analysis of carbohydrate content of transgenic plants

**Figure 9:**
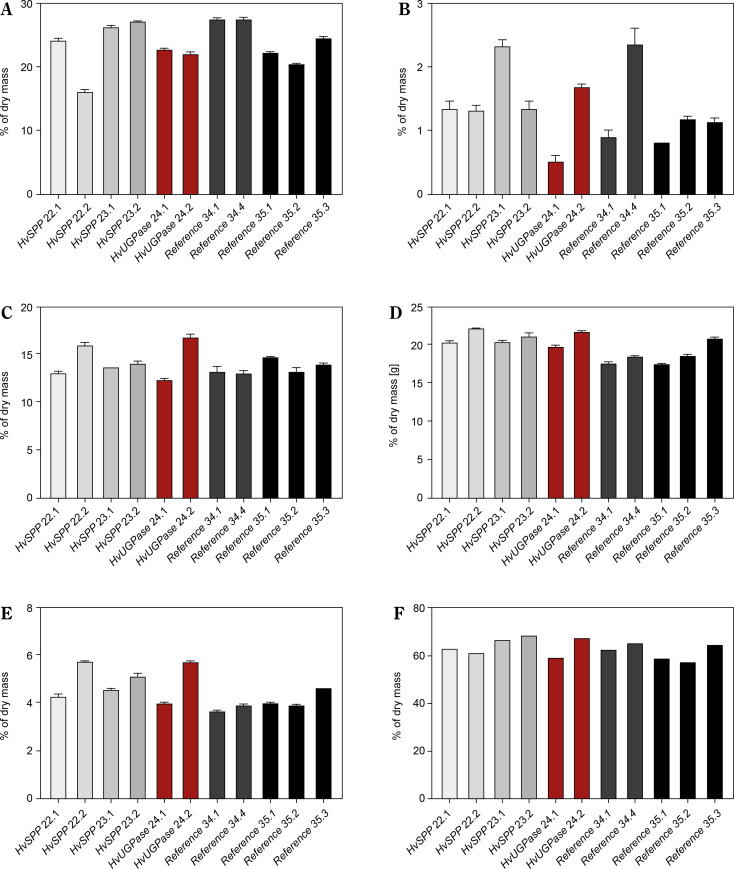
Carbohydrates and lignin composition of the T1 transgenic and reference plants at 69 DAP as % of the dry mass: (**A**) monosaccharides, (**B**) disaccharides, (**C**) hemicellulose, (**D**) cellulose, (**E**) lignin, (**F**) total carbohydrates and lignin. Plants overexpressing *HvUGPase –* ochre bars, plants overexpressing *HvSPP –* grey bars, reference plants – black bars. Data are presented as mean ± standard deviation (SD) from three technical repetitions

The aboveground plant parts collected at 69 DAP were analyzed for carbohydrate and lignin content (Fig. 9). The total sum of mono- and disaccharides, hemicellulose, cellulose, and lignin in *HvUGPase*-and *HvSPP*-over-expressing plants was slightly higher than in reference plants, with differences amounting to 3.2% d.m. for *HvSPP* plants and 1.7% d.m. for *HvUGPase* plants (Fig. 9F).

Monosaccharides were the predominant component of the analyzed plant material, though their content in *HvSPP*-and *HvUGPase*-over-expressing plants was slightly lower than in reference plants (23.5% d.m. and 22.5% d.m., respectively, compared to 24.5% d.m.) (Fig. 9A). Disaccharides contributed less than 2% of the dry mass and had a minor impact on the overall energy value of the plant material. In *HvSPP*-overexpressing plants, disaccharide content was slightly higher than in reference plants, whereas in *HvUGPase*-overexpressing plants, it was slightly lower (1.6% d.m. and 1.1% d.m. vs. 1.3% d.m.) (Fig. 9B). However, for insoluble carbohydrates: hemicellulose and cellulose, as well as lignin, their share in dry mass was higher in plants overexpressing *HvSPP* and *HvUGPase* than in reference plants and amounted to, respectively: for hemicellulose 14.1% d.m. and 14.4% d.m. compared to 13.5% d.m., for cellulose 20.9% d.m. and 20.7% d.m. compared to 18.5% d.m., and for lignin 4.8% d.m. and 4.8% d.m. compared to 4.0% d.m. (Fig. 9C–E). The main difference in carbohydrate composition between plants overexpressing the *HvUGPase* and *HvSPP* genes and the reference plants is most likely due to the higher proportion of lignified material in the dry mass of transgenic plants than in the reference plants, which is associated with the accelerated development of these plants.

The plant material used in these analyses consisted of the entire aboveground portion of the plant after the completion of the generative phase, which closely corresponds to the biomass state utilized for biofuel production. Therefore, these results can be compared with studies analyzing similar material, such as fully developed leaves and stems of sugarcane overexpressing *SPS*, where increased sucrose, fructose, and glucose contents were observed in both organs (Anur et al., 2020). In *Brachypodium distachyon* overexpressing *SPS*, analysis of senescent aboveground biomass revealed a higher sucrose concentration in leaves compared to WT plants, while sucrose concentration in stems remained unchanged (Falter and Voigt, 2016).

In both cases, transgene expression was driven by an ectopic promoter, making it difficult to determine whether the observed phenotypic effects resulted from increased sugar accumulation or enhanced efficiency of the photosynthesis-related sucrose synthesis pathway. Notably, differences were observed between these two examples: *SPS* overexpression increased sucrose content in sugarcane stems but did not produce the same effect in *B. distachyon*. This discrepancy is likely due to sugarcane’s highly efficient sucrose storage mechanism in the stem, a trait absent in *B*. *distachyon*.

### Energy sequestered by transgenic plants overexpressing HvUGPase and HvSPP

The energy value of the plant material from the analyzed plants was measured. This parameter not only represents the upper limit of thermal energy obtained from the complete combustion of one gram of plant material in the presence of oxygen but also allows for the determination of the maximum biofuel energy yield per unit mass, regardless of the biofuel production technology. The plant material used in these analyses comprised the entire aboveground portion of the plant after the generative phase, closely resembling the biomass preferred for energy production.

**Figure 10:**
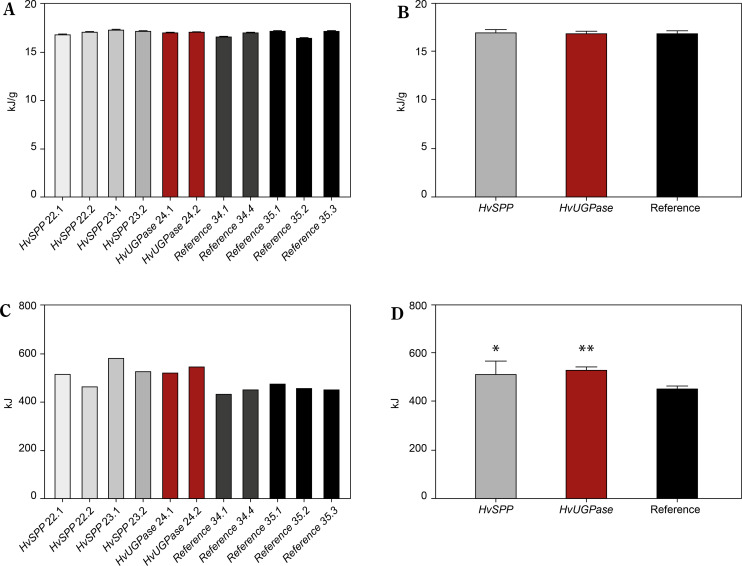
Energy value [kJ/g of d.m.] and energy sequestered in the aerial part of the plants [kJ] of T1 transgenic and reference plants. (**A**) Energy values in transgenic plants overexpressing *HvUGPase* (ochre bars) or *HvSPP* (grey bars), and reference plants (black bars). (**B**) Average energy value of transgenic and reference lines. (**C**) Energy sequestered in the aerial part of the transgenic plants over-expressing *HvUGPase* or *HvSPP*, and reference plants. (**D**) Average energy sequestered in the aerial part of plants overexpressing *HvUGPase* or *HvSPP*, and reference plants. Data are presented as mean ± standard deviation (SD) from three technical repetitions. The asterisks indicate the level of statistical significance, with *p < 0.05, and **p < 0.01

The energy value was slightly higher in plants overexpressing *HvSPP* and *HvUGPase* compared to reference plants, measuring 17.23 kJ/g and 17.16 kJ/g, respectively, versus 16.95 kJ/g in reference plants (Fig. 10A, B). The 1.26–1.65% increase in energy value observed in *HvSPP*- and *HvUGPase*-over-expressing plants was likely due to their higher carbohydrate and lignin content.

Data on energy value and dry mass enabled the calculation of total energy sequestered in the aboveground portion of the plants (Fig. 10C, D). This comparison revealed a clear advantage for *HvSPP*and *HvUGPase*overexpressing plants, which sequestered 14.91–17.55% more energy in their above-ground part of the plant than reference plants over the same vegetation period. This indicates a beneficial effect of overexpression of genes for enzymes of the photosynthetic sucrose synthesis pathway on the energy sequestered in the plant, even if the plant does not have a specific organ responsible for sequestering newly fixed carbon.

## Conclusions

In this study, transgenic *N. tabacum* plants were developed with high *HvUGPase* and *HvSPP* transgene expression in leaves, while transgene expression in roots was 60 to 2400 times lower than in leaves. These findings demonstrate that the *Chrbc-*P promoter enables efficient and leaf-specific transgene expression, even after leaf expansion and in mature or senescent plants.

Here, this leaf-specific expression cassette was used to drive the expression of genes for HvUGPase and HvSPP, two enzymes in the photosynthesis-related sucrose synthesis pathway localized in leaf cells. *N. tabacum* plants overexpressing *HvUGPase* and *HvSPP* under the *ChrbcS1* promoter exhibited faster growth and earlier flowering. These traits contribute to a higher biomass production rate, which is particularly advantageous for energy crops cultivated as aftercrops or in regions with a short growing season, where they do not compete with food production. Transgenic plants with leaf-specific expression of *HvUGPase* or *HvSPP* sequestered 14.9–17.5% more energy in their above-ground parts of the plant than reference plants.

Transgenic modifications that accelerate development and induce earlier flowering represent an alternative strategy to approaches proposed for regions with long growing seasons, where extending the juvenile growth phase is preferred. The modifications of plant metabolism presented in this study are part of the Sormisol Project, which investigated the use of energy crops grown as after crops in the period June/July – September, in the region of Central Europe. Under such conditions, plants have only three to three and a half months to produce biomass. One aspect of the Sormisol Project was to assess how increasing the efficiency of the photosynthesis-related sucrose synthesis pathway impacts plant productivity and biomass production. The obtained effects are beneficial in terms of application but require further studies, especially field tests, to demonstrate their practical usefulness and to elucidate the underlying molecular mechanisms.

Notably, the phenotype of *HvUGPase*- and *HvSPP*- overexpressing plants closely resembles that of plants overexpressing the NAC transcription factor gene, suggesting a potential role for sucrose as a signaling molecule (Grover et al., 2014). The *LlNAC* used as a transgene by Grover et al. was a homolog of the ANAC055 gene from *A. thaliana*, isolated from a cold stress subtractive library. The *N. tabacum* plants with this transgene under the strong, constitutive promoter CaMV S35 accumulated transgene transcripts to high levels, from 158 to 2033.6-fold higher than the actin gene. At the onset of flowering, *LlNAC* transgenic plants were 1.8- to 2.5-fold taller than WT plants and exhibited a 1.25- to 1.73-fold increase in dry weight compared to controls. Additionally, *LlNAC* plants displayed accelerated development, as indicated by earlier bud emergence and a shortened vegetative phase. The authors suggest that the phenotype of *LlNAC* plants is likely due to the activity of this gene as a transcription factor regulating several different biological processes, including cold tolerance, biomass accumulation, and life cycle. Other *NAC* genes have also been identified as master regulatory switches influencing various biological processes (Singh et al., 2021).

The similarity between the phenotypes of *LlNAC* transgenic plants and *HvUGPase*- and *HvSPP*- overexpressing plants indicates that either the LlNAC or its homolog may regulate the photosynthesis-related sucrose synthesis pathway, or changes in the efficiency of this pathway may lead to upregulation of this gene. Therefore, it seems reasonable to hypothesize that the phenotype of *HvUGPase* and *HvSPP* plants, including very diverse effects such as accelerated growth, higher biomass yield, shortened life cycle and earlier entry into the generative phase, results not only from physiological processes induced by increased sucrose supply, but also from the regulatory and signaling role of sucrose.

## Data Availability

The datasets generated during and/or analyzed during the current study are available from the corresponding author upon reasonable request.
